# Reputational Risk, Academic Freedom and Research Ethics Review

**DOI:** 10.1177/0038038515590756

**Published:** 2015-06-25

**Authors:** Adam Hedgecoe

**Affiliations:** Cardiff University, UK

**Keywords:** academic freedom, new public management, research ethics committees

## Abstract

Drawing on scholarship around academic freedom and new public management, this article explores the way in which research ethics committees in UK universities (URECs) can come to exhibit behaviour – common in their US equivalents – that prioritises the reputational protection of their host institution over and above academic freedom and the protection of research subjects. Drawing on two case studies the article shows both how URECs can serve to restrict research that may be ‘embarrassing’ for a university and how, in high profile cases, university management come to use such committees as mechanisms for internal discipline.

It is a truth commonly acknowledged by sociologists that prior ethics review of research – originating as it does in the biomedical sciences – is unsuited for the oversight of social science (particularly qualitative social science), harming and impeding research especially that conducted on sensitive topics and populations. In the UK, while sociological concerns have tended to focus on the research ethics committee (REC) system overseeing research in the National Health Service (NHS) (e.g. Boden et al., 2009; McDonach et al., 2009; Stalker et al., 2004) complaints have also been levelled against the more recent system of University RECs (URECs) (Hammersley, 2009; Holmwood, 2010; Stanley and Wise, 2010). While the growth of URECs can be traced to the 2006 publication of the Economic and Social Research Council’s Research Ethics Framework document (ESRC, 2006), this expansion of ethics review to social science was in tune with broader trends; a review carried out prior to the ESRC Framework suggested that over 80 per cent of UK universities surveyed had some form of ethics committee in place, with a quarter of such committees being set up before 1990 (Tinker and Coomber, 2004).

A key concern on the part of opponents of such review is that it infringes academic freedom by restricting the ability of sociologists and other social scientists to research the topics they deem important. For example, Martin Hammersley argues that, given that the principle of autonomy ‘must surely apply to researchers as well as to the people they study’ (2006: 7) the application of ethics review to the social sciences ‘amounts not only to a bureaucratization of research but also to unwarranted restriction on the freedom of researchers’ (2009: 218; see also Dingwall, 2008). In making these claims, UK-based critics have much in common with colleagues in the USA, where prior ethics review has been presented as an infringement of the first amendment and a clear threat to academic freedom (Hamburger, 2005; Katz, 2007).

However, the relationship between REC review and academic freedom is, at best, unclear. As Eric Barendt (2010: 210) points out, in the UK at least, academic freedom ‘has never been understood to confer positive rights to funding for research or to conduct interviews with, say, health service staff and patients’. Yet opponents of such review might argue that only a minor proportion of social scientific research in the UK is either funded by the ESRC or set in the NHS. Why then should this remaining work be submitted to prior ethical review?

One explanation for the development of URECs and the expansion of ethics review to all social science research (regardless of funder and setting) focuses on the broader context of changes within UK higher education (Hammersley, 2010). This position argues that a key feature of UK universities over the past two decades has been the expansion into higher education of specific ways of organising public sector work, known as ‘new public management’ or NPM. This is a complex ‘set of assumptions and value statements about how public sector organizations should be designed, organized, managed … [where] … the basic idea … is to make [them] … much more “business-like” and “market-oriented”’ (Diefenbach, 2009: 893).

While empirical research underlines how, in a higher education context at least, NPM is more complex than critics often suggest (Deem and Brehony, 2005), there are a generally agreed set of changes to management practice – such as clearer line-management, hierarchical work relations, increasing varieties of audit and league tables and a drive towards externally funded research – that mark out those universities that have undergone NPM (Deem et al., 2007; Kolsaker, 2008; Shore, 2008).

However, the impact of NPM on traditional forms of academic freedom – the freedom to ‘follow a line of research where it leads … [and the] … freedom to teach the truth as we see it’ (Russell, 1993: 18) – is unclear. Exercises such as the UK’s *Research Excellence Framework* may put pressure on academics to publish but aside from quality, the content of those publications remains outside the remit of audit. Even those authors most critical of the impact of NPM on higher education have difficulty producing *empirical evidence* of infringement of academic freedom, relying instead on hypotheticals (Craig et al., 2014). Through the exploration of two case studies this article examines the role of URECs and their potential impact on academic freedom within the context of post-NPM higher education.

For the purposes of this article, a key development in the evolution of NPM is the adoption of risk management practices from the private sector (Lapsley, 2009). While its origin lies in high profile corporate scandals resulting from financial mismanagement – for example, Barings, Enron, WorldCom – Michael Power (2004a: 61) suggests that the growth of risk management as an organisational practice has begun to take a specific form, essentially, that ‘most business people, when asked about the risk which worries them most, will often mention reputation’. The ‘multiplier effect’, whereby apparently minor indiscretions can be seen to indicate larger problems in organisational culture and have large scale repercussions means that ‘reputation risk reflects a new sense of vulnerability … for senior managers … and has created new demands to make reputation “manageable”’ (Power, 2004a: 61; see also Power, 2004b). Carried across from the private sector, the development of new public management means that ‘[i]n the public sector, risk management is a “reputation management strategy”’ (Lapsley, 2009: 16). While the practice of reputation management lacks a widely accepted definition, tending to be seen in terms of public relations or corporate communications (Hutton et al., 2001), it also involves control of internal organisational behaviour as a precursor to managing external reputations (Doorley and Garcia, 2010: 14–16). With the increasing importance of various league tables in higher education, reputation management has, over the past decade or so, become increasingly important to universities (British Council, 2014). Thus the focus of this article is how RECs come to serve as mechanisms for institutional reputation management, and in the process restrict academic freedom.

This article contributes to two specific literatures. The first is the body of work carrying out empirical research on ethics review which has tended to draw on ethnographic fieldwork and has largely focused on the review of biomedical research in both the UK (Dyer, 2004; Hedgecoe, 2012) and other countries (Fitzgerald, 2005; Stark, 2011, 2013). There has also been more limited work focusing on the review of social science (Hedgecoe, 2008) and it is to this latter literature that the current article makes a contribution.

The second contribution is to the small, but growing, literature (e.g. Fowler et al., 2013; Murray, 2013) that takes as its data the results of Freedom of Information (FOI) Act requests (and other typically hidden documents such as accounts of disciplinary hearings), noting that such approaches offer ‘a unique means of studying official information management and public relations activities’ (Walby and Larsen, 2011: 32). The resulting data resemble in many ways Gary Marx’s ‘dirty data’, ‘information which is kept secret and whose revelation would be discrediting or costly in terms of various types of sanctioning … [running] contrary to widely shared standards and images of what a person or group should be’ (Marx, 1984: 78).

The two case studies outlined in this article were chosen opportunistically; they are examples where problems around UREC review became public and where FOI data became available, allowing the opening of these particular regulatory ‘black boxes’. It should not be inferred therefore that the two universities involved are unique in having particular problems around ethics review and the role of management, but rather they are the two most obvious and researchable examples to date.

## URECs, Sex and Institutional Reputation Management^1^

For almost 20 years, newspapers have reported that UK university students have turned to sex work because of reduction in student grants, increases in fees and resulting rising levels of student debt (Duncan, 1997; Herbert, 2006; Telford, 2005), with similar claims being made about students in other countries (e.g. Duval Smith, 2006). Yet at the same time there has been growing resistance on the part of universities in the UK to academic research on the topic of student sex work, to the extent that one researcher in this area claims that:

There is now a growing consensus amongst the community of academics [researching this topic] that our universities are not conducive places for conducting research with students who wish to cash in some of their ‘sexual capital’ … it is evidence that research on this topic is not simply unwelcome, it is actively discouraged. (Roberts, 2010: 13)

Using emails, letters and minutes gathered through a Freedom of Information (FOI) request, this section explores the role of URECs in ‘discouraging’ this kind of research, focusing on the 2004/2005 decision by the Kingston University Faculty of Arts and Social Sciences REC regarding a proposed undergraduate research project. The proposed project looked at some of the factors influencing female students’ decision to enter the sex industry. Because of the sensitive nature of this research topic, this project was referred to the faculty REC by the student’s supervisor, rather than being dealt with by the student project module teaching team. To allow speedy review of the application, it was discussed via email by a subcommittee of the REC before a decision was taken at a meeting.

REC members’ focus was primarily on the potential risks faced by the student researcher: ‘All I think we need to ascertain or build into the project is that the student carrying out the work is safe’ (Anon, 2004a). Another member ‘agree[s] with [name redacted] that the main issue is the safety of the student conducting the experiment. As it is [it is] not clear where the interviews/data collection will take place, except that it will be all over London and vary from person to person’ (Anon, 2004b). The proposed solution to the issue of researcher safety was ‘that the interviews should take place in an (public?) environment that will be safe for both the student and interviewee’ and that in addition ‘[t]he identity of the interviewee must be kept anonymous at all times in the course of the study’ (Anon, 2004c). This last member is relaxed enough about this proposed research to note that:

Normally the ethics committee we [*sic*] would expect to see the consent form and interview schedule to check out how the project is represented to participants and the appropriateness of questions to be asked. *I’m happy to leave the final judgement here to the supervisor if the rest of the committee are [too,] in the interests of speed* … In principle then, I’m happy for the research to go ahead, but would like reassurance on the above points before the project is finally approved. (Anon, 2004c, emphasis added)

Thus at this stage of the process, the REC has given the impression that this project is largely unproblematic with the main ethical focus being the safety of the researcher. Solutions to this have been proposed, and one member has even made clear their willingness *not* to review the consent form, in order to allow the research to proceed in a timely manner. The committee meeting itself took place on the same day as the last supportive email (20 October) and the student’s supervisor was sent a letter noting that: ‘After some discussion and assurances that no harm can come to the interviewer and that potential harm to the interviewees is minimized, this project was cleared’ (Anon, 2004d).

However, soon after getting this approval, the supervisor ‘asked for the full committee to re-examine the project as he was not satisfied that the conditions were warranted’ (Anon, 2011) – what ‘conditions’ are not clear but perhaps around the need to conduct interviews in a public place – and asked for a full review by the whole of the REC. The resulting full committee review on 8 December reiterated the previous points made by the initial review but also introduced a new requirement: that it was ‘[i]mportant to ensure students from Kingston are not included’ (Pickard, 2005). The result of this requirement was to restrict severely the recruitment of participants to this research (Roberts et al., 2007a) and to redirect the researchers’ focus onto a survey of broader student opinions about possible drivers for sex work (Roberts et al., 2007b), an approach that while ‘not ideal because it does not directly capture individual involvement’ does have the advantage of providing a ‘conduit through the maze of research ethics committee requirements’ (Roberts et al., 2007a: 145).

The material released under FOI provides no insight into why this new requirement – that any sex working students should come from another university – was imposed on this project. One obvious explanation is that Kingston University wanted to distance itself from the implication that its own students had to resort to this kind of work, and the possible resulting bad publicity. This interpretation is strengthened by an email from the publicity and press office at Kingston University sent to the student supervisor, referring to the subsequent survey of student *opinions* about possible drivers for student sex work (which *did* access Kingston students):

What worries me is that although I’m sure the research does highlight it [i.e. student sex work] as a problem globally as well as nationally, your research is based on a survey undertaken with [our] University students and it’s the publicity surrounding that which concerns me. The story has hit the international press in India which, as you probably know, is a big market for [the University]. (Roberts, 2010: 14)

As the supervisor and the student note in their discussion of their attempt to get the original project off the ground:

universities themselves show little enthusiasm to cooperate in addressing the question of why their clientele are increasingly turning to employment which entails the provision of sexual services to a paying public. The potential psychological and physical cost to students appears to be of little interest or else *is viewed as a potential source of negative public relations*. (Roberts et al., 2007a: 145, emphasis added)

In this case, even if this was not the actual intention, the effect of the REC’s final decision was, without any obvious increase in protection for research subjects, to protect the university from the potentially scandalous revelation that its students were engaged in sex work.^2^ *Why* the REC chose to impose this restriction (which is not raised in any of the previous email discussions or sub-committee decisions) remains unclear; for example, whether it was suggested by a member of the committee or brought to the committee meeting from the University’s management. Whatever the origin of this decision, the REC served as an instrument of organisational reputation management, preventing the gathering of data that might, when published, harm the University’s interests. Given that the research that *was* completed, which asked about Kingston students’ *second hand* understanding of why students turned to sex work, raised concerns on the part of the University’s press office, the research that was not carried out may well have harmed the University’s reputation. Whether protecting a university’s reputation is a suitable part of a REC’s remit is a different question, of course.

## URECs, Terrorism and Social Control

This second case explores how, when incidents that harm a university’s reputation have already taken place, research ethics review can be employed as a means of internal social control and avoidance of future reputational harm. The background to this centres on the arrest at Nottingham University, on 14 May 2008, of two men, Rizwaan Sabir and Hicham Yezza, under the prevention of terrorism act. Yezza, an administrator at the University, was accused of having a copy of a document called *The Al-Qaida Training Manual*, apparently emailed to him by Sabir, a Master’s student and prospective PhD candidate. Six days later, both men were released without charge: Sabir had asked Yezza to print the document – widely available on the internet from sources such as the US Justice department (from where Sabir had downloaded it) and even Amazon – to help him with his Masters dissertation (on terrorism) and PhD application (Sabir, 2008).

The focus of this section is not the specifics of the decision to arrest Sabir and Yezza but rather the aftermath, both in terms of Sabir’s return to the University (and discussions with administrators) and actions related to Dr Rod Thornton. Thornton, a lecturer in the School of Politics and International Relations, had protested what he saw as unfair treatment of Sabir and Yezza by the University, culminating in a presentation to the British International Studies Association (BISA) in April 2011, outlining his case against the University. The article itself was taken down from the BISA website under threat of legal action from individuals named in it, and Thornton himself was suspended from the University because of the breakdown in working relationships caused by his paper (Jump, 2011).

The following empirical data come from documents, some gathered through FOI and data protection requests, placed on the ‘Unileaks’ website in the wake of Rod Thornton’s suspension.^3^ These include a version of Thornton’s original paper presented at the BISA conference as well as: a copy of the formal HR notes made at Thornton’s disciplinary hearing in 2009; a transcript of a meeting between Rizwaan Sabir (one of the men arrested in the original police operation) and several members of university management; and copies of some of the emails and correspondence referred to in these other documents. While these documents range across a number of issues, this article focuses on two separate issues which highlight both the atypical understanding on the part of senior university management of the purpose of URECs as framed by the ESRC’s rules and the way in which, in such a context of limited understanding, University RECs’ roles can be revised at management request. The first area of interest centres on senior management’s concern over the lack of UREC review of Rizwaan Sabir’s MA dissertation and PhD proposal, and the second, the decision by the Head of School to ask the Politics and International Relations REC to review Rod Thornton’s teaching materials.

A couple of months after the original arrests, the issue of whether Sabir should have sought UREC approval before accessing *The Al-Qaida Training Manual* was raised at the highest level of the University by the incoming Vice Chancellor, who noted in an email:

Now that we have clarity on the nature of the Al Qaeda manual it would be reasonable to ask the question whether access went through the Ethics Committee in the School of Politics and, if not, who gave permission for Mr Sabir to access it. (Greenway, 2008)

This query suggests that it is normal UREC practice to review access to this kind of document, even though it was available from the US Department of Justice website, and at the time was apparently held by the University of Nottingham library. Approving access to published literature, even on such sensitive topics, is not normally regarded as within the remit of research ethics committees. Historically the focus for RECs has been on the ethical issues raised by human subjects research, and this concern remains central to current REC practice. Indeed, the ESRC’s guidelines explicitly state that UREC review is *only* required for ‘[r]esearch proposals involving human participants, as well as research involving more than minimal risk’ (ESRC, 2010: 10). In this context, ‘risk’ is largely defined in terms of the kind of person or group being examined (for example, children, or those who lack the capacity for consent). Even where risk is defined in terms of the kind of document accessed, this revolves around personal or confidential information (ESRC, 2010: 9). A document contained in a university library, or freely available over the internet, would not fall into this category. With no human beings involved in Sabir’s research, the suggestion that some form of ethics approval was required before accessing the *Manual* implies a lack of familiarity with the traditional role and function of URECs or at the very least an idiosyncratic interpretation of the ESRC’s guidelines.

This perspective was held by other members of Nottingham’s senior management. In a meeting with the University’s Registrar and Head of Security held following his release, Sabir was repeatedly pressed over the lack of ‘direction’ or ‘advice’ he had had from his department. When Sabir asked whether he could cite the training manual in his dissertation, the Registrar responded: ‘that’s a question for your supervisors. I guess it’s also a question for the research ethics committee in the School of Politics and International Studies’ (University of Nottingham, 2008), again implying that the REC’s remit extended beyond human subject protection to include oversight of publicly available documents.

The same understanding of the role of RECs apparently existed at the level of the School of Politics and International Relations. On 18 September, following Thornton’s objections to Sabir and Yezza’s treatment, the Head of the School asked a sub-committee of the School’s REC to review Thornton’s *teaching* materials, in the light of the problems caused by Sabir’s accessing of the *Al-Qaida Training Manual*. The reaction of these REC members varied. While two of them ‘sent their responses immediately; the third member did not come back with a reply until 26 September’ (University of Nottingham, 2009: 5) Indeed this member:

Felt that this was an attack on academic freedom … that there were a number of issues and thought this went beyond the remit of the committee. The other two responses saw it enabling teaching, and afforded student and staff protection. (University of Nottingham, 2009: 5)

In an email exchange this reluctant member suggested that:

I am not aware of anything in the remit of Ethics Committee that would warrant a procedure whereby its members become responsible for the approval of module handouts [reading lists]. So I am not really sure what we are supposed to look for here and by what standards or criteria to make a judgement. (cited in Thornton, 2011: 53)

In response, the Head of School explicitly expanded the REC’s remit to include the review of non-research related material: ‘Whilst the vast majority of matters that may require a view in regard to ethics will be research-related, there are cases (such as the present one) where other matters may legitimately fall within the remit of an ethics committee’ (cited in Thornton, 2011: 53). No apparent reference was made to, for example, ESRC guidelines or equivalent documents, so the basis for this expansion is unclear.

The first point to note is that, contra Rod Thornton’s belief that the use of a three member sub-committee contravenes the ESRC’s guidelines (Thornton, 2011: 52), the use of such a sub-committee is explicitly allowed for by the ESRC Ethics Framework (ESRC, 2010). More convincing is Thornton’s suggestion that:

the ESRC … decreed that institutions, in regard to establishing their own research ethics committees, should only use them to consider the effects of research on ‘human participants’. Needless to say, I did not have any ‘human participants’ on my reading lists. (Thornton, 2011: 52)

The obvious question becomes why did the Head of School decide to use the REC to review teaching materials? When asked about the decision to refer Thornton’s teaching materials to the School REC, the Head acknowledged that:

I may have been remiss in not looking for another procedure but I wanted to be in a position to *protect [the] School against any adverse criticism* and the ethics committee existed, was easy and convenient and could act in short time. (University of Nottingham, 2009: 5, emphasis added)

Whatever the potential source of this ‘criticism’, the Head of School, seems to be clearly articulating matters in terms of reputation management. He argues that:

There was a need to reassure others that we were not simply allowing everyone to do as they liked – [there was] need for a control mechanism with the School. So [I] felt that if we looked at [the] reading list within School through our ethics committee this would be an easy way of organising this … It was simply a mechanism so that if we were challenged about reckless teaching or exposed to danger awareness, we had a procedure to ensure that this was not the case. (University of Nottingham, 2009: 5–6)

Thus the decision to refer Thornton’s teaching material to the REC was one of avoiding adverse criticism – the source of which is unclear – in essence, a form of reputation management, the effect of which was to employ the REC as a form of internal social control, an issue raised by Thornton when he asks:

why my reading lists, and mine alone, needed ‘controlling’. Neither one of my courses, and so neither one of my reading lists, was in any way linked to the arrests. I was, moreover, not the only lecturer … who had courses related to terrorism. So why was I being singled out? (Thornton, 2011: 50)

Whether or not referring Thornton’s teaching materials to the School REC was intended as a form of social control, it is clear that in this case an oversight body that was meant to be operating according to the ESRC’s guidelines, could – in the view of managers at several levels of the university – be ‘re-tasked’ to look at materials historically and intellectually outside of its remit in order to prevent further reputational harm.^4^

## Discussion

From a comparative point of view, the use of URECs as mechanisms of organisational reputation management is unsurprising. In the USA, IRBs (Institutional Review Boards) – which serve as the explicit template for URECs (Boulton et al., 2004) – have displayed similar behaviours for over two decades. The title, *Institutional* Review Board, underlines the organisational context of these bodies; they are located within research institutions, answering both to their host and to the Office of Human Research Protection. This is a section of the National Institutes of Health involved in accrediting and overseeing the operation of IRBs, which has the power to punish an institution – for example, by freezing federal funding – should its IRB fail to act properly, provide adequate oversight of research risks or fail to require suitable informed consent materials (for an example of such punishment see Marshall, 1999).

As a result, the ‘lens of analysis for the IRB is risk: risk to human subjects and, by extension, implicitly and sometimes explicitly, risk to the institution of loss of prestige and/or loss of research funding’ (Martin and Inwood, 2012: 9). Within this context of institutional protection, some commentators claim that what counts as a ‘risk’ has expanded beyond kinds of research that might open a university or hospital up to legal sanction or loss of federal research funding. Pragmatically if ‘IRBs are designed to minimize any risk to research institutions’ then ‘one might argue that the complex and bureaucratized process of review offers a serendipitous device to frustrate and deter what is considered a potential threat to an institution’s reputation or access to revenue sources’ (Librett and Perrone, 2010: 738). The normality of this broader, reputational, view of risk – and of IRBs’ role in protecting institutions from it – is underlined by the willingness of IRBs to discuss their actions in these terms. A good example is John Tierney’s description of challenging an IRB about decisions related to one of his PhD students, triumphantly exclaiming to the IRB: ‘“… what you’re saying is that you’re not really worried about human subjects per se, but rather about the potential impact on [this university]”’. However, Tierney’s ‘elation quickly evaporated when, without the least bit of hesitation or shame, they [the IRB] agreed’ (Lincoln and Tierney, 2004: 225). His conclusions that ‘[s]ome IRBs are quite clear and above board that their main concern is protection of the institution from damage’ (Lincoln and Tierney, 2004: 220) is unsurprising, given that a longstanding IRB chair can comfortably and openly state that:

[an] important function served by Institutional Review Boards is to protect the institutional interest … Private universities are largely corporations. The respect for academic freedom within these corporations is desirable, but not indispensable … Institutional Review Boards can forestall the *public image problems* and protect the institution’s reputation by weeding out *politically sensitive studies* before they are approved. (Moss, 2007: 803–804, emphases added)

The position outlined here by Jonathon Moss of the University of Chicago is clearly about more than just protecting institutions’ access to federal funding, but rather broader concerns about research that may embarrass or otherwise damage the image of universities or other research bodies.

The exact nature of this risky, ‘politically sensitive’ research is unclear, but it may involve children, research into people’s sexual preferences and experience (Adler and Adler, 2002) or both (Johnson, 2008), or work that is potentially critical of specific institutions or professions (Librett and Perrone, 2010; Timmermans, 1995). As early as 1983, Murray Levine (1983: 8) warned that IRB review could be used by institutions to undermine ‘research with political implications … [providing] … bureaucratic tactics to force lengthy delays … in the hopes that the proponent of the issue will become discouraged, “cool down” and [have] given up pursuing it’.

The evidence presented in this article suggests that URECs in the UK are quite capable of mirroring the behaviour of their US counterparts, of serving as, in Librett and Perrone’s (2010) elegant turn of phrase, ‘serendipitous device[s] to frustrate and deter potential threats to an institution’s reputation’. However, the drivers for this in the USA are distinct from those in the UK. Unlike US IRBs with concerns about research funding and legal threats, URECs’ role as a mechanism for reputation management stems from the application of NPM to higher education. The end result, though, is much the same: a context where the main goal of risk management becomes protecting the university’s reputation, and where it is perhaps unsurprising that research ethics committees become co-opted by university management to prevent research which might embarrass the institution, to control reading lists which might contain books that may make the university look bad. British-based academics carry out research into children, sex, drugs, criminal behaviours and a whole host of other potentially ‘embarrassing’ topics. If it is the case that ethics review bodies can come to serve the reputational interests of their host institutions (as the US comparison suggests), then it is unsurprising that university research ethics committees have made decisions about such research that are centred on the protection of institutional interests rather than the protection of research participants.

Of course, one does not need URECs to discipline behaviour that threatens universities’ reputations; in 1934 Harold Laski, Professor of Political Science at the London School of Economics (LSE) and ardent socialist was censured for columns in the *Dailey Herald* that infringed a rule requiring LSE teachers ‘to pay proper regard to the reputation of the School when expressing their views’ (Barendt, 2010: 84). And numerous examples exist where academics’ freedom to express their (extramural) political views or (intramural) opinions about the running of universities have run up against management concerns about external reputation. The point is that URECs present a new, and largely unacknowledged, stealthy, mechanism through which management can restrict, not just what academics say to the press or on their blogs, but what research they do in the first place.

URECs, by their very nature, can restrict research and can thus infringe academic freedom. The point is that the *grounds* upon which a UREC restricts research are explicit and limited to the protection of those people enrolled in research. Even critics of the application of prior ethics review to social science acknowledge that ‘academic freedom and the protection of individuals from undue harm are two core principles of the academy, and they are not in conflict with one another’ (Tierney and Corwin, 2007: 392). The question is whether ‘protecting a university from reputational harm’ is a core principle of the academy. If it is not, then restricting academic freedom on these grounds through UREC review of research is illegitimate. While professional codes of conduct for social scientists – for example, those of the British Sociological Association or the Association of Social Anthropologists – do mention obligations towards employers and research sponsors, these are couched in terms of being honest about one’s qualifications or the limits of particular methods. When these guidelines discuss harm, it is with regard to research participants, rather than employers’ reputations, that researchers are told to direct their attention.

In considering the implications of this article, one approach might be to point out the limited nature of only two case studies; perhaps these are the only two instances where universities have used URECs to attempt to manage reputational harm. Perhaps these are outlier examples at institutions where, normally, URECs operate properly, where academic freedom is not overridden in the interests of reputation management. Without other examples, it is hard to say if this is the case or not. However, given that the source of URECs’ role in reputation management lies in widely acknowledged sector wide developments – the rise of NPM in higher education – as well as specific changes noted beyond the sector – the mutation of risk management into reputation management – *and* the extensive comparative evidence available from the USA, it seems reasonable to conclude that these examples represent a larger number of cases, at a range of different universities, where URECs have been used to restrict academic freedom in order to protect institutional reputation.

URECs’ role as mechanisms of reputation management is a direct result of their embedded nature; as bodies set within universities the risk is that they come to protect the interests of those organisations that host them. While the ESRC’s guidelines stress the need for URECs to be ‘free from bias and undue influence from the institution in which they are located’ (ESRC, 2010: 10), the only suggestion to insulate URECs from institutional influence is that ‘RECs include members who are independent of the institution’ (ESRC, 2010: 10). Unfortunately this is the same solution operated in the US IRB system – all IRBs are required to have a non-institutional member – and it is clear that this has not prevented the ‘institutional capture’ of US IRBs. The ESRC’s belief that the ‘independence of RECs is founded on their membership, on strict rules regarding conflict of interests and on regular monitoring of and accountability for their decisions’ (ESRC, 2010: 10) ignores the way in which their accountability tends to be to the management of their host institution, limiting this independence. The cost and speed advantages of devolving prior ethics review down to the level of the university (Tinker and Coomber, 2004) must be set against the way the organisational embedding of URECs provides an opportunity for potentially illegitimate concerns to shape UREC decisions; institutional ethics review bodies, such as IRBs or URECs serve as opportunity structures for management restrictions on academic freedom.

The findings of this article have clear implications for sociologists, their students and for social science research more generally. Given that the context that has driven these developments in ethics review – the rise of NPM in UK higher education – seems well established and part of the mainstream, it is unlikely that universities will, of their own free will, suppress the reputation management role of URECs. As a result, we should expect more cases of these kinds of restrictions of academic freedom, of academics and their students being denied permission to carry out research, not because of risk to participants but because of potential reputational harm to their universities.

Despite this, there are obvious solutions. While critics may claim that these problems require the rejection of the prior ethical review of social science research by URECs altogether, other options present themselves. The first approach is to develop a clear distinction between URECs (at whatever level they operate, whether it be centrally or at the faculty level) and university management, most obviously by restricting membership of such committees to non-managerial staff. What ‘non-managerial’ means is open to debate, but at the simplest level this would exclude deans, heads of school, pro-vice chancellors and registrars (to name but a few) from UREC membership. Such clear separation is common in debates around the role of self-regulatory units within larger organisations (Black, 2001) and avoids the more extreme solution of removing ethics review from individual universities with the use of central or regional, rather than institutional, committees. The second, complementary, approach requires greater transparency around UREC decisions, with minutes of meetings and decisions automatically becoming publically available, and the opening up of committee meetings to observation by members of staff. While this will not necessarily prevent URECs from serving the interests of management in reputation protection, it makes such activity harder to hide and forces such decisions to be articulated in a more open setting. For either of these approaches to become standard within UREC practice requires the ESRC to be willing to return to its ethics framework and introduce further revisions. One objection might be that the ESRC has ‘done enough damage’ by mandating ethics review in the first place, yet the real problem lies in the ESRC’s willingness to introduce a form of institutionalised ethics review that even limited engagement with the literature – and the US experience – would have suggested was problematic and a threat to academic freedom. There is thus, perhaps, an onus on them to remedy the faults in the system they introduced.

## Notes

1. Because FOI releases tend to anonymise names, for consistency and privacy this article continues to anonymise individual academics and staff except where this is impossible (e.g. they are named in the title of documents).

2. One alternative might be that these issues were raised simply because the research was to be carried out by an undergraduate, and that if an experienced academic had proposed the research, these concerns would not have occurred. While impossible to say for sure, the broader problems faced by UK researchers into student sex work generally (regardless of experience) suggests that the student status of the researcher was not the cause of this restriction.

3. At 10 March 2015, this material is linked to via: http://www.scribd.com/doc/57593198/How-a-Students-Use-of-a-Library-Book-Became-a-Major-Islamist-Plot-UNILEAKS-VERSION. It is also available from the author on request.

4. In the summer of 2009, the University of Nottingham set up a ‘module review committee’ to vet all teaching material for references that might be illegal or might incite violence. This in itself raises questions about academic freedom but sits outside of the focus of this article (Newman, 2009).
